# To the R.N. Division

**Published:** 1916-03-11

**Authors:** 


### To the R.N. Division.

No guns beat out his requiem as he lay dying,
Only the whispered silence of the ward, the sighing
Of weary men in pain.

The slow grey morning breaks the night, and faintly falling,
He hears the distant bugle of Death's sentry calling
His tired soul to rest.

No mourning there; only the flag of Empire lying,
The sign and proof of victory for him who, dying,
Won the last height of life.

